# Impact of depersonalization on the course of depression: longitudinal observations from the gutenberg health study

**DOI:** 10.1186/s12888-024-05658-7

**Published:** 2024-03-08

**Authors:** Matthias Michal, Jörg Wiltink, Ana N. Tibubos, Philipp S. Wild, Thomas Münzel, Karl Lackner, Norbert Pfeiffer, Jochem König, Alexander Gieswinkel, Manfred Beutel, Jasmin Ghaemi Kerahrodi

**Affiliations:** 1 Department of Psychosomatic Medicine and Psychotherapy, University Medical Center Mainz, Mainz, Germany; 2 Institute of Clinical Chemistry and Laboratory Medicine, University Medical Center of the Johannes Gutenberg University Mainz, Mainz, Germany; 3 Department of Cardiology I, University Medical Center of the Johannes Gutenberg-University Mainz, Mainz, Germany; 4 Preventive Cardiology and Preventive Medicine, Department of Medicine II, University Medical Center of the Johannes Gutenberg-University Mainz, Mainz, Germany; 5 Department of Ophthalmology, University Medical Center Mainz, Mainz, Germany; 6 Center for Thrombosis and Hemostasis, University Medical Center Mainz, Johannes Gutenberg University Mainz, Mainz, Germany; 7 German Center for Cardiovascular Research (DZHK), University Medical Center Mainz, Partner site Rhine-Main, Mainz, Germany; 8 Institute of Clinical Chemistry and Laboratory Medicine, University Medical Center of the Johannes Gutenberg University Mainz, Mainz, Germany

**Keywords:** Depression, Depersonalization, Derealization, Physical illnesses, Mortality

## Abstract

**Background:**

Symptoms of depersonalization (DP) and derealization (DR) are a risk factor for more severe impairment, non-response to various treatments, and a chronic course. In this study, we investigated the effects of DP/DR symptoms in patients with clinically significant depressive symptoms on clinical characteristics and various outcomes in a representative population-based sample with a 5-year follow-up.

**Methods:**

The middle-aged sample comprised *n* = 10,422 persons at baseline, of whom *n* = 9,301 were free from depressive and DP/DR symptoms. *N* = 522 persons had clinically significant depression (PHQ-9 ≥ 10) and co-occurring DP/DR symptoms, and *n* = 599 persons had clinically significant depression (PHQ-9 ≥ 10) without DP/DR symptoms.

**Results:**

There were substantial health disparities between persons with and without depression. These disparities concerned a wide range of life domains, including lower quality of the recalled early life experiences with the parents, current socioeconomic status, social integration (partnership, loneliness), current social and interpersonal stressors (family, work), functional bodily complaints (e.g., tinnitus, migraine, chest pain), unhealthy lifestyle, and the prevalence of already developed physical diseases. These disparities persisted to the 5-year follow-up and were exceptionally severe for depressed persons with co-occurring DP/DR symptoms. Among the depressed persons, the co-occurrence of DP/DR symptoms more than doubled the risk for recurrence or persistence of depression. Only 6.9% of depressed persons with DP/DR symptoms achieved remission at the 5-year follow-up (PHQ-9 < 5). Depression with and without co-occurring DP/DR worsened self-rated physical health significantly. The impact of depression with co-occurring DP/DR on the worsening of the self-rated physical health status was stronger than those of age and major medical diseases (e.g., heart failure). However, only depression without DP/DR was associated with mortality in a hazard regression analysis adjusted for age, sex, and lifestyle.

**Conclusions:**

The results demonstrated that DP/DR symptoms represent an important and easily assessable prognostic factor for the course of depression and health outcomes. Given the low remission rates for depression in general and depression with DP/DR in particular, efforts should be made to identify and better support this group, which is disadvantaged in many aspects of life.

## Background

Symptoms of depersonalization (DP) and derealization (DR) represent disturbances of perception of the self and the surroundings. The phenomena are characterized by feeling detached or disconnected from the self or environment and can occur from mild to severe pathological states. DP/DR symptoms have a high prevalence of 30–80% in various mental disorders [1–4]. Population-based surveys reported prevalence rates for clinically significant symptoms of DP/DR varying between 11.9% in a large student sample with a mean age of around 16 years [5] and 0.8% in a large population-based community sample with a mean age of 55 ± 10 years [6].

In the DSM-5 [7], DP/DR symptoms are part of the diagnostic criteria of anxiety disorders, cannabis intoxication, the dissociative subtype of posttraumatic stress disorder, of the dissociative features of dissociative identity disorder or constitute a disorder of its one right in depersonalization-derealization disorder. In Borderline personality disorder, dissociative symptoms occur in stress-related situations. In the alternative model of personality disorders, DP/DR symptoms belong to the facet of cognitive and perceptual dysregulation of the psychotic trait domain [7]. Rarely are the symptoms caused by medical diseases such as temporal lobe epilepsy, migraine, vestibular disorder, or specific visual disturbances [8–11]. From a psychodynamic perspective, DP/DR is considered a defense mechanism [12], respectively, a “learned automatic response to reduce or avoid aversive emotional states” [13].

Concerning depressive disorders, symptoms of DP/DR are not part of the current diagnostic criteria in the ICD-10/11 or the DSM-5. However, these symptoms were frequently described as part of major depression in older textbooks of psychiatry or psychological medicine published from ∼1900 to 1960 [14]. The German psychiatrist Petrilowitsch even defined a kind of “dissociative subtype” of major depression (“Estrangement Depression”) akin to the dissociative subtype of PTSD [15]. According to Pertrilowtisch (1956), the distinguishing feature of estrangement depression is the discrepancy between the complaints of the affected persons about their depressiveness, deadness, lack of concentration, and despair on the one hand, and their apparent behavior, which looks almost normal to the environment, on the other hand. Further standard features are complaints about altered bodily sensations and related fears of suffering from a severe physical illness. In addition, complaints about a drop in mental and physical performance, loss of concentration, and rapid exhaustion are almost always present [15]. However, empirical studies on the association of DP/DR symptoms with major depression are sparse. In a small study from Serbia, patients with major depression and severe DP/DR symptoms were more bothered by psychomotor disturbances, insomnia, lack of energy, and poor concentration, and more suicidal ideation was present compared to patients with major depression only [16]. Another small study investigated the impact of DP/DR symptoms in patients with major depression on neuropsychological performance. DP/DR was associated with more pronounced neuropsychological dysfunction [17]. A recent neuroimaging study on depersonalization symptoms in major depression found that DP/DR symptoms were related to reduced connectivity between brain regions proposed to process body-related and autobiographical information [18].

Concerning their prognostic importance, several studies showed that DP/DR symptoms are associated with more severe impairment [6, 19–22] and a higher risk for a chronic course of co-occurring mental disorders or distress [4, 23, 24]. Furthermore, in patients with PTSD [25, 26], Borderline personality disorder [27, 28], obsessive-compulsive disorders [29, 30], and panic disorders, the occurrence of DP/DR symptoms predicted a worse response to psychotherapeutic treatments [13].

Against this background, we aimed to investigate the effects of DP/DR symptoms in patients with clinically significant depression in a large representative population-based sample for clinical characteristics and the outcomes recurrence or persistence and remission of depression, general mental and physical health status, all-cause mortality, and hospitalization. Outcomes that are important for the individual and the healthcare system.

## Methods

The Gutenberg Health Study (GHS) is a large population-based, prospective, observational single-center cohort study in the Rhine-Main-Region, Germany [31]. The local ethics committee and the local data safety commissioner approved the study protocol (reference no. 837.020.07; original vote: 22.3.2007, latest update: 20.10.2015) before the study initiation. Study participants were included after written informed consent. Recruitment and baseline examinations were performed between 2007 and 2012. All study investigations have been conducted per the Declaration of Helsinki and principles outlined in recommendations for Good Clinical Practice and Epidemiological Practice. The inclusion criteria were age 35 to 74 years. Insufficient knowledge of the German language and psychological or physical impairment concerning participation led to exclusion. All GHS participants were randomly selected from local governmental registry offices stratified by sex, age, and residence (urban or rural) at baseline. The adequate recruitment efficacy proportion was 55.5%. A total of 15,010 participants were examined at baseline in 2007–2012. *N* = 12,423 (82.8%) of the GHS baseline participants took part in the 5-year follow-up examination in 2012–2017.

For this analysis, we included persons with the following criteria: Clinically significant depression according to PHQ-9 ≥ 10 or free from depressive symptoms (PHQ-9 < 5) and no symptoms of DP/DR (CDS-2 = 0). We excluded *n* = 3,392 persons with a PHQ-9 score between 5 and 9, *n* = 818 with a PHQ-9 below 10 and a CDS-2 score above 0, and *n* = 378 due to missing data for PHQ-9 or CDS-2. Thus, the baseline sample comprised *n* = 10,422 persons, of which *n* = 9,301 were free from depressive and DP/DR symptoms. Concerning missing data, no imputation was applied, because of the large sample size and the low number of missing values. Participants with missing data were excluded from the respective analysis.

### Data assessment

All participants underwent a standardized assessment, including evaluation of clinical variables, a computer-assisted personal interview, laboratory examinations from a venous blood sample, blood pressure, and anthropometric measurements. The examinations were performed according to standard operating procedures by certified medical technical assistants at the study platform at baseline and the 5-year follow-up [31].

### Computer-assisted personal interview

During the computer-assisted personal interview, participants were asked whether they had ever received a definite diagnosis of any depressive disorder or anxiety disorder from a physician. The presence of coronary artery disease was assessed by the question: ‘Were you diagnosed with a stenosis of your coronary vessels?’ Self-reported heart failure (HF), stroke, peripheral arterial disease (PAD), chronic pulmonary disease (COPD), chronic kidney disease (CKD), and cancer were assessed similarly. Diabetes was defined in individuals with a definite diagnosis of diabetes by a physician or a blood glucose level of ≥ 126 mg/dl in the baseline examination after an overnight fast of at least 8 h or a blood glucose level of > 200 mg/dl after a fasting period of 8 h. Cardiovascular risk factors were defined as follows: Obesity was defined as a body mass index > = 30 kg/m^2^. Smoking was assessed by self-report. Alcohol consumption was measured in grams per day; at-risk consumption of alcohol was defined as daily consumption of ≥ 24 mg for men and ≥ 12 mg for women. Socioeconomic status was defined from 3 (lowest socioeconomic status) to 21 (highest socioeconomic status) based on education, profession, and income [32]. Physical activity was measured with the Short Questionnaire to Assess Health-Enhancing Physical Activity (SQUASH) [33] and described as a metabolic equivalent value.

We used the two-items version of the Cambridge Depersonalization Scale (CDS) to assess depersonalization and derealization. In previous studies, CDS-2 showed high reliability (Cronbach’s alpha = 0.92) and could differentiate patients with clinically significant depersonalization and derealization well from other groups (cut-off of CDS-2 ≥ 3, sensitivity = 78.9%, specificity = 85.7%) and also showed high reliability (Cronbach’s alpha = 0.92) [24, 34]. The response format of the CDS-2 was adopted from the Patient Health Questionnaire (“Over the last 2 weeks, how often have you been bothered by any of the following problems? /Not at all = 0/Several days = 1/More than half the days = 2/Nearly every day = 3”). For the assessment of depression, the Patient Health Questionnaire (PHQ-9) was applied. Clinically significant depression was defined by a score ≥ 10. This cut-off has an 81% sensitivity and 82% specificity regarding the detection of depressive disorder [35], and PHQ-9 < 5 determined the absence of clinically relevant depressive symptoms and remission, respectively [36]. The history of any suicide attempt was assessed at the 5-year follow-up by self-report. Generalized anxiety was assessed with the two-item short form of the GAD-7 (Generalized Anxiety DisorderGAD–2 Scale). A sum score of 3 or more (range 0–6) out of these two items indicates generalized anxiety with good sensitivity (86%) and specificity (83%) [37]. Social anxiety was determined by scoring six or higher on the Mini-Social Phobia Inventory [38]. In addition, the distressed personality was assessed with the DS14 [39, 40].

Medication was recorded using the Anatomical Therapeutic Chemical (ATC) Classification System. For this analysis, we included antidepressants (N06A), antipsychotics (N05A), hypnotics/anxiolytics (N05C/N05B), and opioid painkillers (N02A). Psychiatric or psychotherapeutic treatment was assessed by the number of consultations during the last month. The answers were dichotomized into yes versus no.

The following medical conditions were assessed by the question, “have you been treated in the last two years because of tinnitus, migraine headache, and back pain. Further, chest pain and irregular heartbeat have been determined by the questions “have you ever experienced pain or discomfort in the chest” and “do you know times when the heart beats irregularly”.

The current self-rated mental and physical health was determined by the question, “how would you rate your current physical or mental condition?”. The response format has four levels (1 = very good = 1; 2 = good; 3 = less good; 4 = poor).

The following common strains were assessed and rated on a 5-point Likert scale (1 = No, does not apply; 2 = Yes, applies, but has not burdened; 3 = Yes, applies, has burdened me little; 4 = Yes, true, has burdened me moderately; 5 Yes, true, has burdened me heavily): Troubles with the boss, troubles with colleagues, family troubles and “frequent loneliness, too few social contacts”. The common strains were determined as no if the score was 1 or 2, and yes if it was ≥ 3.

The ultra-short screening version of the Recalled Parental Rearing Behavior questionnaire (FEE-US) was used to assess childhood adversities [41, 42]. The scale tracks the remembered parenting behavior of father and mother in terms of the following three categories: Rejection & punishment, emotional warmth, and control & overprotection.

### Statistical analysis

We described the data as absolute and relative proportions for categorical data, means and standard deviations (SD) for continuous variables with approximately normal distribution, and median with quartiles (Q1/Q3) if not fulfilling this criterion. Baseline data were compared by Chi-square test for dichotomous variables, t-tests for mean and Wilcox rank sum test for more skewed data. The associations of DP/DR symptoms with various dependent variables and outcomes were analyzed by logistic, linear, hazard, and proportional odds regression models with 95%-confidence intervals (95%CI) and univariate and multivariate models. Due to the large sample size, considering effect estimates, p-values should be interpreted cautiously. Cohen’s d was specified for continuous variables to evaluate the group differences better. We calculated the analyses with R version 4.2.1 (R Core Team, 2022).

## Results

There were *n* = 522 (5%) persons with clinically significant depression (PHQ-9 ≥ 10) and co-occurring DP/DR symptoms (CDS-2 > 0). *N* = 599 (6.4%) persons had clinically significant depression (PHQ-9 ≥ 10) without DP/DR symptoms (CDS-2 = 0). Depressed persons with either DP/DR symptoms or without DP/DR differed significantly (with *p* < 0.05) from persons without depression in all variables of Table 1. When comparing depressed individuals with DP/DR with depressed persons without co-occurring DP/DR symptoms, meaningful differences emerged for the following psychosocial variables: Depressed persons with DP/DR had more symptoms in all distress scales (PHQ-9 [d = 0.61], GAD-2 [d = 0.53], social anxiety, distressed personality, poorer physical [d = 0.29] and mental health [d = 0.43], were more burdened by interpersonal problems in the family, with colleagues, loneliness, lived less often in a partnership and they were more frequently taking antidepressants and having consulted psychiatrists during the last month. Concerning common physical symptoms, they endorsed more lifetime episodes of chest pain.

**Table 1:** Psychosocial baseline characteristics

	Not depressed (PHQ-9 < 5) and no DP/DR (CDS-2 = 0) (*n* = 9,301)	Depressed (PHQ-9 ≥ 10)		Cohen’s d^§^	Test for depression with DP/DR versus depression only
		no DP/DR (CDS-2 = 0) (*n* = 599)	DP/DR (CDS-2 ≥ 1) (*n* = 522)		
Age in years, mean (SD)	55.3 (11.2)	53.0 (10.3)	52.5 (10.0)	-0.05	0.44
Sex, % (n)	45.2% (4200/9301)	61.9% (371/599)	58.8% (307/522)		0.30
Social economic status, mean (SD)	13.38 (4.47)	12.08 (4.24)	11.76 (4.23)	-0.08	0.21
Living in a partnership, % (n)	84.5% (7858/9301)	72.1% (432/599)	64.6% (337/522)		**0.0068**
**Mental distress**					
Depression severity (PHQ-9), median (Q1/Q3)	2.00 (1.00/3.00)	11.00 (10.00/13.00)	13.00 (11.00/16.00)	0.61	*p* < 0.0001
DP/DR: CDS-2, median (Q1/Q3)	0 (0/0)	0 (0/0)	2.00 (1.00/2.00)	2.53	*p* < 0.0001
Clinically significant DP/DR: CDS-2 > = 3, n (%)	0% (0/9301)	0% (0/599)	11.3% (59/522)		*p* < 0.0001
Anxiety: GAD-2, median (Q1/Q3)	0 (0/1.00)	2.00 (1.00/3.00)	3.00 (2.00/4.00)	0.53	*p* < 0.0001
Clinically significant anxiety (GAD-2 > = 3), % (n)	0.8% (74/9269)	38.2% (227/595)	56.7% (295/520)		*p* < 0.0001
Clinically significant social anxiety, % (n)	2.8% (259/9282)	24.4% (146/599)	42.8% (223/521)		*p* < 0.0001
Distressed personality, % (n)	13.9% (1291/9271)	51.8% (309/597)	65.9% (344/522)		*p* < 0.0001
History of Depression, % (n)	5.4% (499/9294)	37.8% (226/598)	58.2% (302/519)		*p* < 0.0001
History of anxiety, % (n)	3.6% (337/9293)	19.1% (114/598)	33.1% (172/520)		*p* < 0.0001
History of suicide attempt, % (n)	1.1% (85/7659)	9.6% (43/448)	14.4% (55/381)		**0.040**
**Health status and common functional complaints**					
Tinnitus, % (n)	6.4% (592/9292)	12.0% (72/599)	14.6% (76/522)		0.22
Migraine headache, % (n)	4.9% (451/9297)	13.2% (79/598)	14.4% (75/520)		0.60
Back pain, % (n)	71.6% (151/211)	67.4% (161/239)	61.0% (1294/2121)		0.36
Chest pain, % (n)	26.1% (2430/9296)	50.9% (305/599)	60.5% (316/522)		**0.0014**
Irregular heartbeat, % (n)	12.1% (1124/9295)	31.2% (187/599)	35.1% (183/522)		0.18
Current state of physical health, mean (SD)	1.95 (0.55)	2.63 (0.76)	2.85 (0.80)	0.29	**< 0.0001**
Current state of mental health, mean (SD)	1.81 (0.53)	2.78 (0.71)	3.09 (0.70)	0.43	**< 0.0001**
**Interpersonal difficulties**					
Troubles with the boss, % (n)	4.0% (368/9211)	18.6% (109/587)	22.2% (114/513)		0.13
Troubles with colleagues, % (n)	3.2% (291/9216)	13.4% (79/589)	18.1% (93/515)		**0.038**
Family troubles, % (n)	8.8% (815/9239)	39.5% (234/592)	48.5% (252/520)		**0.0030**
Frequent loneliness, % (n)	1.5% (140/9252)	19.7% (116/589)	35.9% (186/518)		*p* < 0.0001
**Medication and Treatment**					
Psychiatric treatment*, % (n)	0.2% (14/9296)	2.2% (13/599)	5.7% (30/522)		**0.0027**
Psychotherapeutic treatment*;% (n)	0.4% (36/9296)	4.2% (25/599)	5.2% (27/522)		0.48
Antidepressants, % (n)	2.4% (219/9199)	18.7% (111/595)	27.3% (142/521)		**0.00074**
Antipsychotics, % (n)	0.4% (38/9199)	2.5% (15/595)	3.6% (19/521)		0.30
Hypnotics/Anxiolytics, % (n)	1.1% (103/9199)	5.9% (35/595)	7.7% (40/521)		0.23
Opioid pain killers, % (n)	0.9% (83/9199)	3.9% (23/595)	4.8% (25/521)		0.46

Continuous variables are described by mean values with standard deviation in brackets (SD) or median values with 1st and 3rd quantile in brackets (Q1 / Q3) if they are skew. Discrete variables are described through relative and absolute frequencies. Baseline data were compared by Chi-square test for dichotomous variables, t-tests for normally distributed continuous variables, and Wilcox rank sum test for more skewed continuous data

Note: In the comparison of not depressed versus depressed persons, all variables of this Table differed significantly

*Psychiatric or psychotherapeutic treatment during the last month

^§^Cohen’s for continuous variables

Table 2 shows the three groups’ prevalence of common medical diseases and biomarkers. Depressed persons had higher levels of CRP, higher blood pressure, a worse LDL/HDL ratio, and peripheral artery disease, heart failure, and COPD were more frequent. In comparing depressed persons as a function of DP/DR, there was only a worse LDL/HDL ratio. Concerning lifestyle factors, depressed persons were significantly more often smoking, obese, and less physically active (Table 3). Non-depressed persons had a higher rate of at-risk alcohol consumption. Comparing depressed persons as a function of DP/DR, persons with DP/DR had a higher smoking rate.

**Table 2:** Medical disease and biomarkers

	Not depressed (PHQ-9 < 5) and no DP/DR (CDS-2 = 0) (*n* = 9,301)	Depressed (PHQ-9 ≥ 10)		Cohen’s d^§^	Test for depression with DP/DR versus depression only
		no DP/DR (CDS-2 = 0) (*n* = 599)	DP/DR (CDS-2 ≥ 1) (*n* = 522)		
Hypertension, % (n)	50.2% (4664/9300)	48.8% (292/598)	46.4% (241/519)		0.44
Systolic blood pressure in mm Hg, mean (SD)	132.3 (17.6)	128.1 (16.1)	127.6 (15.9)	-0.03	0.61
LDL/HDL ratio, mean (SD)	2.58 (0.92)	2.56 (0.94)	2.70 (1.00)	0.14	**0.017**
C-reactive protein (CRP) in mg/dl, median (Q1/Q3)	1.50 (0.50/3.00)	2.00 (0.93/4.10)	1.90 (0.89/4.20)	0.00	0.77
Coronary artery disease, % (n)	4.0% (367/9187)	5.4% (32/589)	5.8% (30/514)		0.79
Peripheral artery disease, % (n)	2.8% (256/9233)	4.2% (25/590)	6.8% (35/513)		0.063
Stroke, % (n)	1.6% (148/9272)	3.0% (18/594)	2.5% (13/517)		0.72
Heart Failure, % (n)	1.0% (95/9297)	2.8% (17/599	2.5% (13/521)		0.85
CKD, % (n)	0.8% (78/9299)	1.3% (8/599)	1.1% (6/522)		0.36
COPD, % (n)	3.9% (366/9297)	9.3% (56/599)	10.9% (57/522)		0.43
Diabetes, % (n)	8.4% (782/9301)	10.9% (65/599)	11.9% (62/522)		0.64
Cancer, % (n)	8.5% (791/9294)	8.7% (52/598)	10.2% (53/521)		0.41

Continuous variables are described by mean values with standard deviation in brackets (SD) or median values with 1st and 3rd Quantil in brackets (Q1 / Q3) if they are skew. Discrete variables are described through relative and absolute frequencies. Baseline data were compared by Chi-square test for dichotomous variables, t-tests for normally distributed continuous variables, and Wilcox rank sum test for more skewed continuous data

Note: In the comparison of participants who are depressed with those not depressed, all variables differed significantly except for hypertension, CKD, and cancer

^§^Cohen’s for continuous variables

**Table 3:** Lifestyle factors

	Not depressed (PHQ-9 < 5) and no DP/DR (CDS-2 = 0) (*n* = 9,301)	Depressed (PHQ-9 ≥ 10)		Cohen’s d^§^	Test for depression with DP/DR versus depression only
		no DP/DR (CDS-2 = 0) (*n* = 599)	DP/DR (CDS-2 ≥ 1) (*n* = 522)		
Current smoking, % (n)	17.7% (1645/9293)	24.4% (146/599)	31.5% (164/521)		**0.0090**
Physical activity, median (Q1/Q3)	7.05 (4.84/9.21)	7.43 (5.28/9.48)	7.05 (5.06/10.03)	-0.01	0.87
Obesity, BMI > = 30, % (n)	23.1% (2151/9299)	33.0% (197/597)	31.4% (164/522)		0.61
Alcohol abuse*, % (n)	23.2% (2155/9301)	20.9% (125/599)	19.0% (99/522)		0.45

Continuous variables are described by mean values with standard deviation in brackets (SD) or median values with 1st and 3rd Quantil in brackets (Q1 / Q3) if they are skew. Discrete variables are described through relative and absolute frequencies. Baseline data were compared by Chi-square test for dichotomous variables, t-tests for normally distributed continuous variables, and Wilcox rank sum test for more skewed continuous data

Note: In the comparison of participants who are depressed with those not depressed, all variables differed significantly

* daily consumption of ≥ 24 mg for men and ≥ 12 mg for women

^§^Cohen’s for continuous variables

### Recalled parental rearing behavior

Depressed patients had worse recalled parental rearing behavior with both parents than non-depressed patients (Table 4). The effect sizes ranged from d = 0.29 to d = 0.52. The most prominent effects were found for rejection and punishment by the mother [d = 0.52] and by the father [d = 0.53]. Moreover, in the group of depressed patients, DP/DR was associated with worse recalled parental rearing behavior compared to depressed persons without DP/DR. The differences between these groups were in the range of small effects and were highest for rejection and punishment by the mother [d = 0.24] and by the father [d = 0.27], as well as control and overprotection by the mother [d = 0.23].

**Table 4:** Recalled parental rearing behavior

	Not depressed (PHQ-9 < 5) and no DP/DR (CDS-2 = 0) (*n* = 9,301)	Depressed (PHQ-9 ≥ 10)		Cohen’s d^§^	Test for depression with DP/DR versus depression only
		no DP/DR (CDS-2 = 0) (*n* = 599)	DP/DR (CDS-2 ≥ 1) (*n* = 522)		
Emotional warmth (Mother), mean (SD)	2.77 (1.39)	2.43 (1.57)	2.19 (1.58)	-0.16	**0.024**
Emotional warmth (Father), mean (SD)	2.02 (1.44)	1.73 (1.48)	1.44 (1.36)	-0.21	**0.0041**
Control & overprotection (Mother), median (Q1/Q3)	1.00 (0/2.00)	1.00 (0/2.00)	1.00 (0/2.00)	0.23	**0.0012**
Control & overprotection (Father), median (Q1/Q3)	0 (0/1.00)	1.00 (0/2.00)	1.00 (0/2.00)	0.17	**0.0033**
Rejection & punishment (Mother), median (Q1/Q3)	0 (0/1.00)	0 (0/1.00)	1.00 (0/2.00)	0.24	**0.00023**
Rejection & punishment (Father), median (Q1/Q3)	0 (0/1.00)	0 (0/1.00)	1.00 (0/2.00)	0.27	**0.0021**

Continuous variables are described by mean values with standard deviation in brackets (SD) or median values with 1st and 3rd Quantil in brackets (Q1 / Q3) if they are skew. Discrete variables are described through relative and absolute frequencies. Baseline data were compared by Chi-square test for dichotomous variables, t-tests for normally distributed continuous variables, and Wilcox rank sum test for more skewed continuous data

Note: In the comparison of participants who are depressed with those not depressed, all variables differed significantly

^§^Cohen’s for continuous variables

### 5-year follow-up rates by group

The two depressed groups had a significantly lower follow-up rate regarding the follow-up assessment. The respective rates for the three groups were 85.3% for the non-depressed group, 77.3% for the depressed group without DP/DR, and 76.8% for the depressed persons with DP/DR.

### 5-year follow-up: depression outcome

At the five-year follow-up (Table 5), persons with depression and DP/DR symptoms had a substantially worse outcome than the comparison group of depressed persons only: 59.7% versus 39.7% were in the range of clinically significant depression. In the age and sex-adjusted logistic regression analysis with the outcome PHQ-9 ≥ 10, persons with depression and DP/DR had a 63.11-fold risk compared to the non-depressed group for being depressed five years later (OR 63.11, 95% CI, 48.91–81.44, *p* < 0.001). The odds ratio for the only depressed group was OR 27.50 (95% CI, 21.56–35.08, *p* < 0.001). This picture was not changed significantly after additional adjustment for the following baseline variables: SES, hypertension, coronary artery disease, peripheral artery disease, stroke, heart failure, chronic kidney disease, chronic obstructive pulmonary disease, current smoking, alcohol abuse, obesity, physical activity, intake of antidepressants, antipsychotics, hypnotics/anxiolytics, and opioid pain killers: The risk for depression with DP/DR was OR 55.59 (95% CI, 40.78–75.78, *p* < 0.001) versus for depression only OR 24.19 (95% CI, 18.10–32.33, *p* < 0.001). Among the long list of baseline covariates, only the following were associated with the outcome PHQ-9 ≥ 10: Sex (OR 1.44, 95% CI, 1.12–1.84, *p* = 0.0042), age per year (OR 0.99, 95% CI, 0.97-1.00, *p* = 0.044), SES (OR 0.96, 95% CI, 0.93–0.99, *p* = 0.0088), smoking (OR 1.35, 95% CI, 1.02–1.79, *p* = 0.036), heart failure (OR 4.74, 95% CI, 1.73–13.01, *p* = 0.0025), and intake of antidepressants (OR 1.64, 95% CI, 1.12–2.39, *p* = 0.011). Among the depressed persons, the occurrence of DP/DR symptoms more than doubled the risk for recurrence or persistence of depression (OR 2.30, 95% CI, 1.50–3.51, *p* < 0.001).

**Table 5:** Mental health outcomes at the 5-year follow-up

	Not depressed (PHQ-9 < 5) and no DP/DR (CDS-2 = 0) (*n* = 9,301)	Depressed (PHQ-9 ≥ 10)		Cohen’s d^§^	Test for depression with DP/DR versus depression only
		no DP/DR (CDS-2 = 0) (*n* = 599)	DP/DR (CDS-2 ≥ 1) (*n* = 522)		
PHQ-9 ≥ 10, % (n)	2.2% (169/7792)	39.7% (182/458)	59.7% (233/390)		*p* < 0.0001
PHQ-9 < 5, % (n)	78.4% (6109/7797)	15.9% (73/459)	6.9% (27/391)		*p* < 0.0001
PHQ-9 sum score, median (Q1/Q3)	3.00 (1.00/4.00)	8.00 (6.00/12.00)	11.00 (7.88/14.00)	0.42	*p* < 0.0001
GAD-2 ≥ 3, % (n)	2.3% (177/7736)	26.9% (122/454)	42.4% (165/389)		*p* < 0.0001
GAD-2 sum score, median (Q1/Q3)	0 (0/1.00)	2.00 (1.00/3.00)	2.00 (2.00/3.00)	0.38	*p* < 0.0001
CDS-2 sum score, median (Q1/Q3)	0 (0/0)	0 (0/0)	1.00 (0/2.00)	0.71	*p* < 0.0001
CDS-2 ≥ 3, % (n)	0.2% (15/7773)	2.9% (13/456)	13.1% (51/388)		*p* < 0.0001
Physical health, mean (SD)	2.02 (0.56)	2.51 (0.71)	2.64 (0.69)	0.20	*p* = 0.0041
Mental health, mean (SD)	1.90 (0.59)	2.60 (0.74)	2.69 (0.72)	0.13	*p* = 0.051

Continuous variables are described by mean values with standard deviation in brackets (SD) or median values with 1st and 3rd Quantil in brackets (Q1 / Q3) if they are skew. Discrete variables are described through relative and absolute frequencies. Baseline data were compared by Chi-square test for dichotomous variables, t-tests for normally distributed continuous variables, and Wilcox rank sum test for more skewed continuous data

Note: In the comparison of participants who are depressed with those not depressed, all variables differed significantly

^§^Cohen’s for continuous variables

Concerning the linear outcome PHQ-9 sum score at the 5-year follow-up, the fully adjusted linear regression model demonstrated that each point increase on the CDS-2 predicted a 0.76-point increase in the PHQ-9 (β = 0.76, 95% CI: 0.53–0.99, *p* < 0.001). The corresponding betas for baseline PHQ-9 and GAD- 2 sum scores were (β = 0.54, 95% CI, 0.52–0.57, *p* < 0.001) and (β = 0.28, 95% CI, 0.21–0.35, *p* < 0.001). The impact of the CDS-2 score was more than twice that of anxiety (GAD-2).

Regarding the achievement of remission at the 5-year follow-up as determined by a PHQ-9 score < 5, the fully adjusted logistic regression model revealed depressed persons had a meager chance of remission: Depressed persons with DP/DR (OR 0.03, 95% CI: 0.02–0.04, *p* < 0.001); without DP/DR (OR 0.07, 95% CI: 0.05–0.09, *p* < 0.001). Indeed, only 6.9% of depressed persons with DP/DR symptoms achieved remission versus 15.9% of the only depressed group (Table 5).

### Self-rated physical and mental health outcome at the 5-year follow-up

As shown in Tables 1 and 2, the three groups had significant self-rated health disparities, with the worst health status in depressed persons with DP/DR symptoms. These disparities continued to the 5-year follow-up (Table 5), although the self-rated health improved in all groups slightly. We performed a proportional odds regression model with the outcome worsening of health status at the 5-year follow-up adjusted for baseline score of self-rated mental of physical health status and the covariates age, sex, BMI, smoking, at-risk alcohol consumption, physical activity, hypertension, coronary artery disease, peripheral artery disease, stroke, heart failure, chronic kidney disease, COPD, diabetes, cancer, obesity, intake of antidepressants, antipsychotics, hypnotics/anxiolytics, opioid pain killer. The fully adjusted model revealed that depression with or without DP/DR symptoms strongly impacted mental and physical health. For worsening of mental health from baseline to follow-up, the odds ratios were: depression only (OR 2.77, 95% CI, 2.17–3.52, *p* < 0.0001) and depression with DP/DR (OR 2.10, 95% CI, 1.65–2.67, *p* < 0.0001). For physical health, the numbers were: depression without DP/DR (OR 2.10, 95% CI, 2.08–2.67, *p* < 0.0001) and depression with DP/DR (OR 2.70, 95% CI, 2.08–3.50, *p* < 0.0001). The impact of depression on the worsening of the self-rated physical health status was stronger than those of age (per year OR 1.01, 95% CI, 1-1.02, *p* = 0.00040) and those of severe medical conditions (e.g., heart failure (OR 1.36, 95% CI, 0.75–2.44, *p* = 0.30); peripheral artery disease (OR 1.67, 95% CI, 1.2–2.31, *p* = 0.0023), chronic kidney disease (OR 1.85, 95% CI, 0.97–3.43, *p* = 0.058); chronic obstructive pulmonary disease (OR 1.31, 95% CI, 1.00-1.69, *p* = 0.045).

### Outcome hospitalization

There were *n* = 3,628 hospitalizations without a diagnosis of a primary mental disorder in the follow-up period. In the age and sex-adjusted proportional hazard regression analysis, neither depression with DP/DR nor without DP/DR was associated with these hospitalizations. However, the duration of the first event of hospitalization was significantly prolonged by the severity of DP/DR and depression, as demonstrated in the multiple Poisson regression analysis (adjusted for age, sex, SES, and the medical diseases of Table 2). Each point increase in the CDS-2 increased the duration of inpatient treatment days by 17% (RR 1.17, 95%CI, 1.15–1.19, *p* < 0.0001). The respective numbers for depression (PHQ-9) and anxiety (GAD-2) were (RR 1.03, 95%CI, 1.032–1.039, *p* < 0.0001) and (RR 0.87, 95%CI, 0.86–0.88, *p* < 0.0001).

There were *n* = 137 hospitalizations with a leading mental disorder diagnosis. In the age and sex-adjusted hazard regression analysis, depression with and without DP/DR increased the risk for these hospitalizations (“depression with DP/DR” HR 6.27, 95%CI, 4.11–9.58, *p* < 0.001); “depression without DP/DR” (HR 4.89 95%CI, 3.14–7.59, *p* < 0.001). Concerning the overall duration of these hospitalizations, the Poisson regression analysis (adjusted for age, sex, and SES) showed that only depression was positively associated with the duration of the first hospitalization, whereas anxiety and DP/DR were inversely correlated (PHQ-9, RR 1.10, 95%CI, 1.09–1.11, *p* < 0.001; GAD-2, RR 0.79, 95%CI, 0.77–0.80, *p* < 0.001; CDS-2, RR 0.93, 95%CI, 0.90–0.95, *p* < 0.001).

### Outcome mortality

In the follow-up period, *n* = 227 persons died. Depression with or without DP/DR was associated with an increased mortality risk in the age and sex-adjusted hazard regression analysis (depression only, HR 2.34, 95%CI, 1.47–3.72, *p* < 0.001; depression with DP/DR, HR 2.38, 95%CI, 1.42–3.98, *p* < 0.001). The impact of depression was equivalent to around nine years of life (age per year HR 1.10, 95%CI 1.08–1.12, *p* < 0.001). After additional adjustments for baseline variables BMI, current smoking, at-risk alcohol consumption, and physical activity, only depression without DP/DR was still associated with mortality (HR 2.00, 95%CI, 1.14–3.50, *p* = 0.015), whereas the association with depression and DP/DR disappeared (HR 1.56, 95%CI 0.79–3.08, *p* = 0.20). The HRs for the adjusted variables were: female sex (0.61, 95%CI, 0.44–0.85, *p* = 0.0038); age per year (HR 1.10, 95%CI 1.08–1.12, *p* < 0.001); BMI (HR 1.04, 95%CI 1.01–1.07, *p* = 0.0036); current smoking (HR 2.62, 95%CI 1.84–3.74, *p* < 0.001); at-risk alcohol consumption (HR 0.92, 95%CI 0.65–1.31, p < = 0.64); physical activity (HR 0.96, 95%CI 0.91-1.00, *p* = 0.076).

### Temporal stability of DP/DR symptoms

In the group of persons with depression and DP/DR, the CDS-2 score at baseline correlated with the CDS-2 score five years later, with *r* = 0.4, indicating moderate temporal stability. Of *n* = 522 persons with baseline CDS-2 > 0, a proportion of 77% were still (or again) bothered by DP/DR symptoms at the follow-up, and of *n* = 9900 persons without baseline DP/DR symptoms, only 6% endorsed at least one DP/DR symptom five years later.

## Discussion

We aimed to investigate the effects of DP/DR symptoms in persons with clinically significant depression for clinical characteristics and outcomes in a large representative middle-aged population-based sample. We reported huge health disparities between persons with and without depression. These disparities concerned a wide range of life domains, including lower quality of the recalled experiences with the parents, current SES, social integration (partnership, loneliness), current social and interpersonal stressors (family, work), functional bodily complaints (e.g., tinnitus, migraine, chest pain), unhealthy lifestyle, and the prevalence of already developed physical diseases (e.g., COPD, Heart Failure), which often occur as a consequence of a long-lasting unhealthy lifestyle and psychosocial stressors [43]. Moreover, these disparities persisted to the 5-year follow-up and were exceptionally severe for depressed persons with co-occurring DP/DR symptoms.

The co-occurrence of DP/DR symptoms significantly deteriorated the prognosis of depression. The risk of being still or again depressed five years later was more than twice as high in the group of depressed persons with DP/DR. This effect was mainly explained by DP/DR symptoms as demonstrated by the linear regression analyses (corrected for baseline severity of depression and anxiety). Only 7% of the depressed persons with DP/DR symptoms achieved remission. However, the remission rate of the depressed comparison group without DP/DR was also alarmingly low at 16%. These remission rates - defined as PHQ-9 < 5 - are extremely low in comparison with literature reviews, reporting that around 10–37% of depressed patients have a chronic course [44–47]. Even if a far more restrictive cut-off of PHQ-9 ≥ 10 was applied, the rates for a chronic course in our sample would still be very high, with 40% for depression without DP/DR and 60% for depression with DP/DR. These huge differences might result from the sample characteristics. We investigated a community or real-world sample, not patients in a controlled treatment trial. It might be that a large proportion of our sample did not use mental health care adequately. Indeed, only 3% of depressed individuals had seen a psychiatrist during the last month, and only 4.6% had a psychotherapist. However, due to our study’s insufficient coverage of real healthcare usage, we could not further clarify this hypothesis. Nonetheless, this explanation is still astonishing because mental health care is fully covered by health insurance in Germany, and the Rhine-Main region has a high density of psychiatrists and psychotherapists in private practice as well as mental health hospitals.

So far, there has been scarce empirical literature on the importance of DP/DR for the prognosis of depressive disorders. There are only unsystematic reports from the older literature showing that the co-occurrence of DP/DR is associated with longer depressive phases, poor response to drug treatment, poor response to electroconvulsive treatment, and sleep deprivation [4]. One reason for the negative prognostic impact of DP/DR might be that depressive disorders with DP/DR have a stronger link with attachment trauma, as reflected in the worse quality of the recalled rearing experiences, and thus might lead to lower responsiveness to different treatment modalities. Adverse childhood experiences are considered a risk factor for a more chronic and treatment-resistant course of major depression [48–51]. DP/DR symptoms are a specific sequelae and defensive response to attachment trauma [52, 53]. Further, transient DP/DR often occurs in persons with personality disorders, which were found to predict a chronic course and poor treatment response to depression [46, 54].

Regarding the self-rated health status, depressed persons with DP/DR perceived their mental and physical health worse than those of depressed persons without DP/DR. The worse mental health status was also reflected in more severe symptom scale scores and more events in the psychiatric history (previous diagnosis of depression and anxiety disorder, suicide attempts). Regarding the prognosis of depression, it is known that greater severity of the mental health condition, characterized by, e.g., greater comorbidity of mental disorders, particularly anxiety and personality disorders, is a risk factor for chronicity [45, 47].

The more significant impairment of the physical well-being in the persons with DP/DR and depression was not so easily understandable. The frequency of chronic physical illnesses or functional complaints was the same in the two depressed groups, except that lifetime chest pain was reported more frequently in persons with DP/DR. Although physical health status improved slightly in the depressed groups from baseline to follow-up, the physical health status was still the worst in persons with DP/DR. Moreover, DP/DR was an important factor in the deterioration of the physical health status, having a stronger influence than age or severe physical illnesses (e.g., heart failure). However, at the same time, depression without DP/DR was more robustly associated with mortality than depression with DP/DR. In persons with DP/DR, the effect of depression on mortality was mainly due to smoking. Concerning hospitalization due to medical diagnoses other than mental disorders, depression showed no robust association. However, especially DP/DR symptoms prolonged the inpatient time significantly. It might be hypothesized that these effects in the realm of physical health are due to several reasons: Firstly, patients often assume physical causes for DP/DR like in other functional disorders. Secondly, patients with DP/DR often have higher symptom complexity and are more challenging to manage (lack of social support, more interactional difficulties). Concerning the effect of DP/DR on mortality, we speculate that DP/DR symptoms might buffer the toxic impact of depression. A small study showed that DP/DR severity correlated negatively with noradrenergic output [55]. In contrast, major depression is associated with higher norepinephrine excretion levels, which are supposed to hurt the cardiovascular system and may shorten the lifespan [56].

Despite several strengths of the study, like large sample size, comprehensive data collection, standardized assessment of health outcomes, and multi-disciplinary approach, this study has several limitations. First, the reliance on self-report measures constrains clinical conclusions, as we have no information about the diagnostic status of the self-rated symptoms. As outlined in the introduction, DP/DR symptoms can occur along with many mental disorders (e.g., borderline personality disorder, dissociative subtype of PTSD, anxiety disorders), so other factors linked with the DP/DR symptoms might explain the associations of this study. Thus, it is important to remember that we used DP/DR symptoms as a transdiagnostic marker and not as a clinical entity for the interpretation of the results. Therefore, our results cannot be interpreted in terms of a dissociative subtype of depression, but they may inspire further research in this area. Second, the GHS participants were recruited voluntarily, possibly leading to self-selection bias. In addition, the GHS was conducted in a specific region of Germany, predominantly of European ancestry, which may not represent the full diversity of the population. This may limit the generalizability of the findings to other ethnic or racial groups. Finally, in the 5-year follow-up, there were significantly more drop-outs in the group of depressed persons, which could introduce bias.

## Conclusions

Our results show that DP/DR symptoms represent an important and easily assessable prognostic factor for the self-rated health status and the course of depression. Given the low remission rates for depression in general and depression with DP/DR in particular, efforts should be made to identify and better support this group, which is disadvantaged in many aspects of life.

## Data Availability

Written informed consent from GHS study participants does not allow public access to the data. According to the ethics vote, access to the local database is possible anytime upon request. This concept was developed with the local data protection officer and the ethics committee (local ethics committee of the Rhineland-Palatinate Medical Association, Germany). Interested scientists can make their requests to the Gutenberg Health Study Steering Committee (e-mail: info@ghs-mainz.de).

## Abbreviations

BMI: body mass index
CDS-2: two-item version of the Cambridge Depersonalization Scale
COPD: chronic pulmonary disease
CRP: c-reactive protein
DP: depersonalization
DR: derealization
GAD-2: two-item anxiety screener
HDL: high-density lipoprotein
HR: hazard ratio
LDL: low-density lipoprotein
OR: odds ratio
PHQ-9: depression module of the patient health questionnaire
PTSD: posttraumatic stress disorder
SD: standard deviation
